# Comparative Efficacy of Janus Kinase Inhibitors (JAKis) for Ulcerative Colitis in Japanese Regional Healthcare Facilities: A Real-World Database Study

**DOI:** 10.7759/cureus.97500

**Published:** 2025-11-22

**Authors:** Yuki Itoi, Yu Hashimoto, Takashige Masuo, Tomoyuki Masuda, Kyoko Marubashi, Atsuo Iwamoto, Masanori Sekiguchi, Kazuhiro Takahashi, Yuko Kimura, Yoko Hachisu, Shota Tomaru, Keigo Sato, Hirohito Tanaka, Hiroko Hosaka, Shiko Kuribayashi, Yoji Takeuchi, Toshio Uraoka

**Affiliations:** 1 Department of Gastroenterology and Hepatology, Gunma University Graduate School of Medicine, Maebashi, JPN; 2 Department of Gastroenterology, Isesaki Municipal Hospital, Isesaki, JPN; 3 Department of Gastroenterology, National Hospital Organization Takasaki General Medical Center, Takasaki, JPN; 4 Department of Gastroenterology and Hepatology, Kusunoki Hospital, Fujioka, JPN; 5 Department of Gastroenterology, Public Tomioka General Hospital, Tomioka, JPN; 6 Department of Gastroenterology and Hepatology, Maebashi Red Cross Hospital, Maebashi, JPN; 7 Department of Gastroenterology, Haramachi Red Cross Hospital, Higashiagatsuma, JPN; 8 Department of Gastroenterology, National Hospital Organization Shibukawa Medical Center, Sibukawa, JPN; 9 Department of Gastroenterology, Saiseikai Maebashi Hospital, Maebashi, JPN

**Keywords:** adverse reactions, drug-related side effects, janus kinase inhibitors, treatment outcome, ulcerative colitis

## Abstract

Background and aim: Tofacitinib (TOF), filgotinib (FIL), and upadacitinib (UPA) are approved Janus kinase inhibitors (JAKis) for ulcerative colitis (UC), but real-world comparative data remain limited. This study assessed their efficacy, persistence, and safety in regional Japanese hospitals.

Methods: We retrospectively analyzed 148 UC treatment sessions (TOF, 60; FIL, 53; UPA, 35). The primary outcome was clinical remission (partial Mayo score ≤1 with rectal bleeding score 0). Secondary outcomes included clinical response, steroid-free remission, and treatment persistence. Adverse events and biologics (Bio)/JAKi subgroup data were collected.

Results: At week 8, clinical remission rates were similar for TOF (46%), FIL (44%), and UPA (46%). At week 24, FIL had the highest remission rate (61%) vs. TOF (46%) and UPA (41%), a trend that continued at week 48 (59%, 41%, 44%). FIL also showed the highest remission in both Bio/JAKi-naïve and failure sessions at weeks 24 and 48. UPA had the highest clinical response at week 8 (67%). TOF showed stable remission over time (46%, 46%, 41%). Steroid-free remission was more frequent with FIL at weeks 24 (61%) and 48 (59%) than with TOF (46%, 41%) or UPA (35%, 33%). Persistence was similar across agents through week 24. Safety profiles aligned with prior data, though rare events such as *Pneumocystis jirovecii* pneumonia and interstitial pneumonia were noted.

Conclusions: FIL showed favorable remission rates, especially in Bio/JAKi-naïve patients, and remained effective after prior failures, suggesting broad applicability. UPA and TOF showed similar remission rates in our cohort.

## Introduction

Ulcerative colitis (UC) is a chronic inflammatory disease with multifactorial pathogenesis that involves genetic predisposition, epithelial barrier dysfunction, immune dysregulation, and environmental factors [1,2]. Advances in treatment have introduced biologics (Bio) and Janus kinase inhibitors (JAKis), which target the JAK-STAT signaling pathway, a critical mediator of immune responses and cellular processes [3-5]. The JAK family includes JAK1, JAK2, JAK3, and tyrosine kinase 2 (TYK2), each playing distinct roles in cytokine receptor signaling.

Currently, three JAKis are available for use in patients with UC: tofacitinib (TOF), a pan-JAK inhibitor, and the selective JAK1 inhibitors filgotinib (FIL) and upadacitinib (UPA) [6]. TOF and UPA are globally approved, while FIL is widely used in Europe, the United Kingdom, and Japan. The clinical efficacy of TOF was demonstrated in the OCTAVE trials, with remission induction rates ranging from 16.6% to 18.5%; the rates of remission maintenance in the 5 and 10 mg groups were 34.3% and 40.6%, respectively, both of which were significantly higher than the rate for placebo [7,8]. In the SELECTION trial, FIL achieved remission induction rates of 26.1% in Bio-naïve patients and 11.5% in Bio-experienced patients; the remission maintenance rate was 37.2% in the 200 mg group [9,10]. The efficacy of UPA was demonstrated by the U-ACHIEVE and U-ACCOMPLISH trials, where remission induction rates ranged from 26% to 34% and remission maintenance rates were as high as 52% [11].

Selecting which JAKi to use in an individual patient remains challenging. Real-world evidence has emerged, but interstudy differences in patient characteristics and definitions of efficacy hinder direct comparisons [12-18]. Network meta-analyses have suggested that UPA has a faster onset of action [19-21], but these analyses rely on indirect comparisons and lack patient-level adjustments, limiting their applicability. Although real-world studies comparing clinical efficacy between the different JAKis have been published, they were conducted in high-volume centers, which might limit their generalizability in broader clinical practice settings [22-24]. This study aimed to compare real-world outcomes of TOF, FIL, and UPA for UC in regional Japanese hospitals. The primary objective was clinical remission at weeks 8, 24, and 48; secondary objectives were clinical response, steroid-free remission, treatment persistence, and adverse events, with subgroup analyses by prior Bio/JAKi exposure (naïve vs. failure). Prior Japanese multicenter comparisons were mainly from tertiary inflammatory bowel disease centers, which may limit generalizability to routine settings.

## Materials and methods

Study design and population

This retrospective study examined a cohort of patients with UC who visited any of 11 hospitals in the Gunma Prefecture in Japan and were registered in our inflammatory bowel disease database between August 2018 and August 2024. Gunma Prefecture, located approximately 100 kilometers northwest of Tokyo, has a population of approximately 1.9 million people. UC was diagnosed based on the criteria of the Research Committee on Inflammatory Bowel Disease in Japan [25]. The study was approved by the Institutional Ethics Committee at Gunma University Hospital (approval number: HS2024-299). Each site abstracted prespecified variables into a standard Excel template and uploaded the file to a secure, access-controlled repository. A shared data dictionary and operational definitions were used across centers; no central adjudication was performed. Because this was a retrospective registry, visit timing and some clinical evaluations were at the discretion of treating physicians, with no prospective standardization. This registry operates under an IRB-approved data-use policy; patient-level data are not publicly shareable outside collaborating institutions.

**Figure 1:**
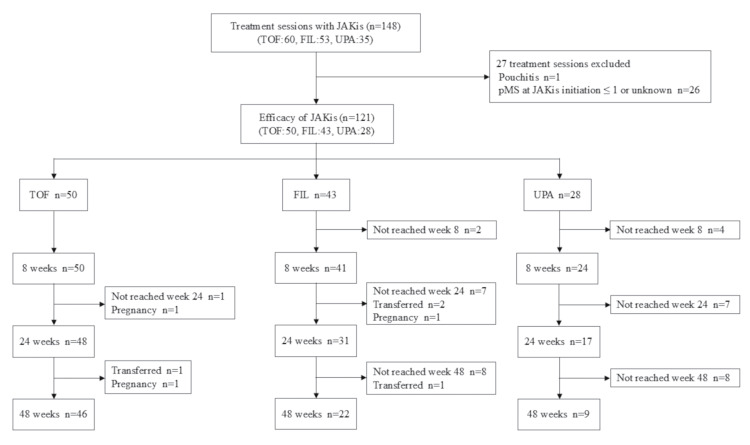
Study flowchart JAKis: Janus kinase inhibitors; TOF: tofacitinib; FIL: filgotinib; UPA: upadacitinib

The unit of analysis in the study was the treatment session rather than the individual patient. A session was defined as the initiation of treatment with a specific JAKi and its subsequent administration until its discontinuation. Treatment in which a patient was switched from one JAKi to another was considered to comprise multiple sessions. Bio/JAKi-naïve patients were defined as those with no prior exposure to biologics or JAKis. Bio/JAKi failure was defined as inadequate response or intolerance to at least one biologic or JAKi. Among the 1535 UC patients who were registered in the database during the study period, 148 JAKi treatment sessions were identified (TOF, 60; FIL, 53; UPA, 35). One session was excluded because of a pouchitis diagnosis, and 26 were excluded because of an unknown partial Mayo score (pMS) or a score ≤1 at the time of initiation. Therefore, 121 sessions were eligible for efficacy analysis, including 50 with TOF, 43 with FIL, and 28 with UPA. Outcomes were evaluated eight, 24, and 48 weeks after treatment initiation. The nominal time points of eight, 24, and 48 weeks were selected a priori to mirror common assessment windows in UC trials and real-world reports: week 8 for induction and weeks 24 and 48 for mid-term and 12-month maintenance, respectively. This facilitates comparability with pivotal randomized controlled trials (RCTs) and prior Japanese multicenter studies. Sessions that did not reach the predefined observation periods were excluded from the corresponding time-point analyses. Additionally, seven sessions were excluded from follow-up owing to pregnancy (TOF, 2; FIL, 1; UPA, 0) or patient transfer to another institution (TOF, 1; FIL, 3; UPA, 0). A study flowchart is shown in Figure 1.

Baseline characteristics

The following data were retrospectively collected: age, sex, duration of UC, disease extent based on the Montreal classification [26], disease activity according to the pMS [27], and endoscopic activity according to the Mayo endoscopic subscore. Baseline laboratory data included serum albumin, C-reactive protein, and hemoglobin. The details of concomitant drugs and history of treatment at baseline were also recorded for each session.

Outcomes and definitions

The primary outcome was clinical remission, defined as a pMS ≤1 with rectal bleeding score 0. Secondary outcomes included clinical response (defined as ≥2-point reduction from baseline pMS), steroid-free remission (defined as clinical remission without systemic corticosteroid use (oral or parenteral) at that time; topical/rectal corticosteroids were not considered systemic), and cumulative JAKi persistence rate. We also compared biomarker values between sessions that achieved clinical remission and those that did not. All potential JAKi-related adverse events were recorded. Subanalyses of treatment sessions in Bio/JAKi-naïve patients and in patients who had failed previous Bio/JAKi treatment were also performed. Clinical remission, clinical response, and steroid-free remission were physician-assessed using the pMS documented at routine outpatient visits; patient-reported instruments were not used. To evaluate JAKi efficacy, sessions involving patients who underwent total proctocolectomy and those in patients with pMS ≤1 at treatment initiation were excluded. Sessions in which JAKis were discontinued because of pregnancy and those which were lost to follow-up because of patient transfer were excluded from observation at that time. JAKi selection and decisions regarding treatment discontinuation adhered to the Ministry of Health, Labour and Welfare of Japan treatment guidelines, which reflect approved indications and dosing regimens [25]; however, final decisions were made at the discretion of the treating physician. JAKi persistence was calculated until the date of drug discontinuation or the date of the last hospital visit.

Statistical analyses

Categorical variables are presented as frequencies with percentages and were compared using Fisher's exact test. Continuous data are presented as medians with interquartile range (IQR) unless otherwise stated and were compared using the Mann-Whitney U test. JAKi persistence was analyzed using the Kaplan-Meier method and compared using the log-rank test. P<0.05 was considered significant. Statistical analyses were performed using EZR version 1.61 (Saitama Medical Center, Jichi Medical University, Saitama, Japan), which is a graphical user interface for R (The R Foundation for Statistical Computing, Vienna, Austria) [28]. Owing to limited sample size and events, we did not perform propensity score or multivariable adjustments to minimize overfitting; available-case analyses were used, and missingness is disclosed in table footnotes.

## Results

Baseline characteristics

Baseline characteristics for the treatment sessions are shown in Table 1. Sixty-three percent of treatment sessions were conducted in men. Sixteen sessions involved patients with a history of prior JAKi use: none in the TOF group, three in the FIL group (TOF, 2; UPA, 1), and 13 in the UPA group (TOF, 6; FIL, 7). The median age at treatment initiation in the TOF, FIL, and UPA groups was 44, 42, and 48 years, respectively. The median disease duration was five, six, and two years, respectively. The median pMS was 6, 5, and 5, respectively. Pancolitis-type UC was most prevalent in the TOF group (86%), followed by the UPA (63%) and FIL (59%) groups. The median laboratory values were similar across groups. Nearly 90% of all sessions were conducted in patients who had received systemic corticosteroids. Immunomodulator use was more frequent in the TOF group (85%) than in the FIL (59%) and UPA (41%) groups. The proportion of Bio/JAKi-naïve sessions was highest in the FIL group.

**Table 1:** Baseline characteristics JAKi: Janus kinase inhibitor; IQR: interquartile range; TNF: tumor necrosis factor Percentages may not sum to 100% owing to missing values; available-case denominators are shown.

Characteristics	Tofacitinib (n=60)	Filgotinib (n=53)	Upadacitinib (n=35)	P-value
Age at JAK initiation, y, median (IQR)	44 (32, 55)	42 (27, 57)	48 (36, 65)	0.21
Sex				
Male, n (%)	38 (63.3)	33 (62.3)	22 (62.9)	1
Female, n (%)	22 (36.7)	20 (37.7)	13 (37.1)	
Disease duration, y, median (IQR)	5 (2, 9)	6 (2, 11)	2 (0, 6)	0.0062
Disease extent, n (%)				
Proctitis	0 (0)	1 (1.9)	2 (5.7)	0.013
Left-sided colitis	4 (11.1)	20 (37.7)	7 (20.0)	
Pancolitis	31 (86.1)	31 (58.5)	22 (62.9)	
Partial Mayo score, median (IQR)	6 (5, 7)	5 (3, 6)	5 (3, 7)	0.0043
Mayo endoscopic subscore, median (IQR)	2 (2, 3)	2 (2, 3)	3 (2, 3)	0.21
1, n (%)	3 (8.1)	4 (14.3)	2 (9.1)	
2, n (%)	17 (45.9)	12 (42.9)	6 (27.3)	
3, n (%)	17 (45.9)	11 (39.3)	14 (63.6)	
Albumin (g/dL), median (IQR)	3.8 (3.5, 4.2)	4.0 (3.7, 4.2)	3.6 (2.9, 4.0)	0.002
C-reactive protein (mg/dL), median (IQR)	0.33 (0.25, 0.87)	0.20 (0.08, 0.63)	0.48 (0.12, 1.90)	0.12
Hemoglobin (g/dL), median (IQR)	12.2 (10.9, 14.1)	13.1 (11.8, 14.3)	12.5 (10.2, 14.3)	0.27
Concomitant drugs at baseline, n (%)				
5-Aminosalicylic acid	42 (75)	33 (62.3)	16 (47.1)	0.027
Systemic corticosteroid	15 (26.8)	15 (28.3)	13 (38.2)	0.5
Topical corticosteroid	7 (12.5)	3 (5.7)	1 (2.9)	0.27
History of treatment at baseline				
Previous systemic corticosteroid, n (%)	56 (96.6)	45 (88.2)	30 (93.8)	0.25
Previous immunomodulator, n (%)	49 (84.5)	30 (58.8)	13 (40.6)	<0.001
Previous biologics/JAKis, n (%)	34 (58.6)	20 (37.7)	23 (67.6)	0.013
Anti-TNF-α agent, n (%)	27 (46.6)	16 (30.2)	16 (47.1)	
Infliximab	17 (29.3)	9 (17.0)	9 (26.5)	
Adalimumab	10 (17.2)	6 (11.3)	1 (2.9)	
Golimumab	4 (6.9)	4 (7.5)	6 (17.6)	
Vedolizumab	10 (17.2)	5 (9.4)	7 (20.6)	
Ustekinumab	4 (6.9)	7 (13.2)	2 (5.9)	
Tofacitinib		2 (3.8)	6 (17.6)	
Filgotinib	0 (0)		7 (20.6)	
Upadacitinib	0 (0)	2 (3.8)		
Number of biologic/JAKi failure, n (%)				
0	24 (41.4)	33 (62.3)	11 (32.4)	
1	18 (31.0)	9 (17.0)	11 (32.4)	
2	16 (27.6)	5 (9.4)	8 (23.5)	
3	0 (0.0)	6 (11.3)	4 (11.8)	

JAKi efficacy

**Figure 2:**
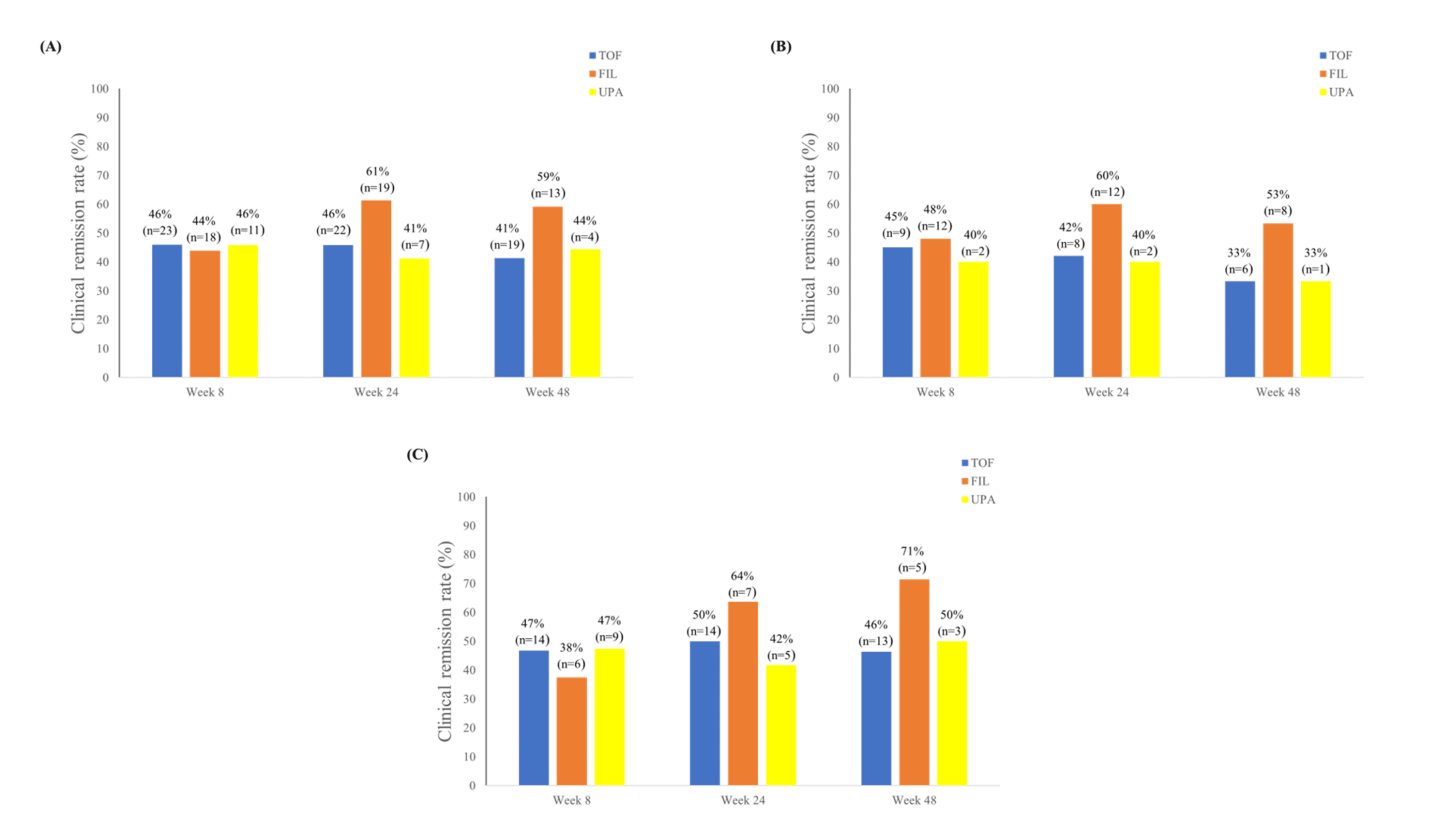
Bar charts showing the clinical remission rates for tofacitinib, filgotinib, and upadacitinib at weeks 8, 24, and 48 (A) Overall and (B) among treatment sessions in Bio/JAKi-naïve patients and (C) among sessions in Bio/JAKi failure patients. TOF: tofacitinib; FIL: filgotinib; UPA: upadacitinib; JAKi: Janus kinase inhibitor; Bio: biologics

**Figure 3:**
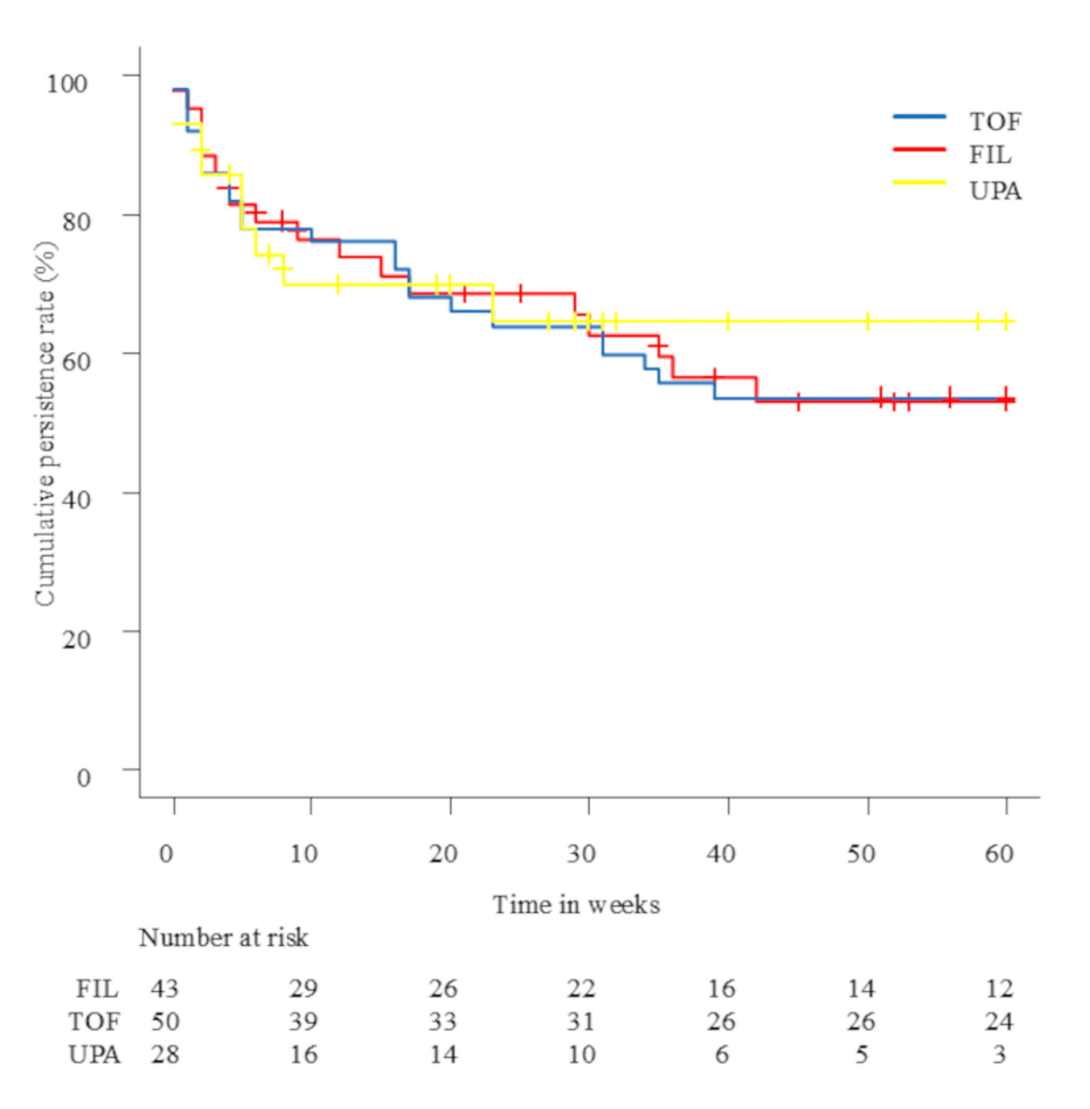
Kaplan-Meier curves of cumulative persistence for tofacitinib, filgotinib, and upadacitinib over time The number at risk is displayed below the x-axis for each group at specific time points. TOF: tofacitinib; FIL: filgotinib; UPA: upadacitinib

The median observation period in the TOF, FIL, and UPA groups was 137.5, 49, and 30.5 weeks, respectively. At week 8, the clinical remission rates were comparable among the groups, ranging from 44% to 46%. At week 24, the remission rate was higher in the FIL group (61%) than in the TOF (46%) and UPA (41%) groups. This trend continued at week 48, with corresponding rates of 59%, 41%, and 44%, respectively (between-group p=0.98, p=0.29, and p=0.40 at weeks 8, 24, and 48; Figure 2A). The clinical response rate at week 8 was 54% for TOF, 51% for FIL, and 67% for UPA (p=0.46); responses remained broadly similar at weeks 24 and 48 (p=0.58 and p=0.60). Steroid-free remission followed a similar trend: at week 8, approximately 42% of patients in each group achieved steroid-free remission. By week 24, the remission rate was higher in the FIL group (61%) than in the TOF (46%) and UPA (35%) groups. This trend persisted at week 48, with corresponding rates of 59%, 41%, and 33%, respectively (p=1.00, p=0.19, and p=0.29 at weeks 8, 24, and 48). In subgroup analyses, FIL had the highest remission rates in both Bio/JAKi-naïve and failure sessions at weeks 24 and 48 (naïve: p=1.00, p=0.57, and p=0.54 at weeks 8, 24, and 48; failure: p=0.85, p=0.61, and p=0.52) (Figure 2B-2C). Clinical remission rates were mostly comparable regardless of previous Bio/JAKi use. The cumulative persistence rates were similar across the three agents at week 8 (78-79%) and week 24 (64-69%). At week 60, however, persistence was higher for UPA (65%) than for the other two (p=0.95) (Figure 3). JAKi discontinuation occurred owing to lack of primary response, loss of response, adverse events, pregnancy, and voluntary treatment cessation. Baseline concentrations of albumin, C-reactive protein, and hemoglobin did not significantly differ between sessions achieving remission and those that did not.

Safety profile

Among all 148 treatment sessions, 26 adverse events were observed: 17 in the TOF group (28.3%), four in the FIL group (7.5%), and five in the UPA group (14.3%). Details are shown in Table 2. Discontinuation because of an adverse event occurred in nine TOF treatment sessions (15%); the specific reason was herpes zoster in three sessions and hepatotoxicity, sinusitis, coronavirus disease 2019, oral candidiasis, kidney dysfunction, and headache in one session each. Among the FIL sessions, four (7.5%) were discontinued owing to an adverse event (hepatotoxicity, urinary tract infection, skin infection, and interstitial pneumonia). Similarly, four UPA sessions (11.4%) were discontinued because of *Pneumocystis jirovecii *pneumonia (PCP), elevated β-D-glucan, sore throat, and acne.

**Table 2:** Adverse events, n (%) COVID-19: coronavirus disease 2019

Event classification	Tofacitinib 17 (28.3)	Filgotinib 4 (7.5)	Upadacitinib 5 (14.3)
Herpes zoster	5 (8.3)	0 (0)	0 (0)
Hepatotoxicity	3 (5.0)	1 (1.9)	0 (0)
Dyslipidemia	2 (3.3)	0 (0)	0 (0)
Sinusitis	2 (3.3)	0 (0)	0 (0)
COVID-19 infection	1 (1.7)	0 (0)	0 (0)
*Pneumocystis jirovecii* pneumonia	0 (0)	0 (0)	1 (2.9)
Elevated β-D-glucan	0 (0)	0 (0)	1 (2.9)
Cytomegalovirus colitis	0 (0)	0 (0)	1 (2.9)
Pharyngitis	1 (1.7)	0 (0)	0 (0)
Oral candidiasis	1 (1.7)	0 (0)	0 (0)
Urinary tract infection	0 (0)	1 (1.9)	0 (0)
Skin infection	0 (0)	1 (1.9)	0 (0)
Kidney dysfunction	1 (1.7)	0 (0)	0 (0)
Interstitial pneumonia	0 (0)	1 (1.9)	0 (0)
Headache	1 (1.7)	0 (0)	0 (0)
Sore throat	0 (0)	0 (0)	1 (2.9)
Acne	0 (0)	0 (0)	1 (2.9)
Thrombosis	0 (0)	0 (0)	0 (0)
Cancer	0 (0)	0 (0)	0 (0)
Death	0 (0)	0 (0)	0 (0)

## Discussion

This study has two key findings: (1) FIL demonstrated higher remission and steroid-free remission rates than TOF and UPA at 24 and 48 weeks, and (2) the proportion of Bio/JAKi-naïve sessions was highest in the FIL group. FIL's superior remission rates were consistent regardless of previous Bio/JAKi use, highlighting its wide applicability. Although TOF and UPA were less effective than FIL, they demonstrated stable efficacy regardless of Bio/JAKi use.

Recent studies conducted at specialized centers have consistently highlighted the efficacy of UPA in overall comparisons. In a study of 168 UC patients, the eight-week clinical response and remission rates were higher for UPA than for FIL [23]. Akiyama et al. reported remission rates of 73% for UPA, 51% for FIL, and 46% for TOF (median follow-ups of 49, 56, and 112 weeks, respectively) [24]. However, the proportion of Bio/JAKi-naïve sessions was higher in our FIL cohort (62%) than in the other studies' cohorts (55% and 48%, respectively), which probably explains the discrepancy in remission rates. The SELECTIONLTE study demonstrated FIL's sustained efficacy, with remission rates reaching 80% at 144 weeks among completers and improving over time in non-responders [10]. These findings align with our results, emphasizing FIL's potential for durable effectiveness. Additionally, our data from non-specialized hospitals provide a broader view of clinical practice, capturing diverse patient characteristics and responses, further highlighting FIL's applicability across varied settings.

FIL demonstrated superior remission rates at weeks 24 and 48 in both Bio/JAKi-naïve sessions and Bio/JAKi failure sessions. Other studies have also reported evidence of its efficacy in Bio-naïve patients [9,16,23]. Additionally, our findings suggest that FIL can be effective in Bio/JAKi failure sessions; however, further studies are needed to confirm. Two previous studies have also reported that treatment history has no significant effect on FIL's efficacy [16,24]. In our study, TOF achieved consistent remission rates of 47%, 50%, and 46% at weeks 8, 24, and 48 among Bio/JAKi failure sessions, reflecting the durable effects reported in the OCTAVE trials [7,8]. While TOF is reliable for sustained remission in relatively stable cases, UPA's rapid symptom control and strong efficacy in refractory UC make it suitable for severe, active cases that require immediate intervention. These findings align with those of prior studies, including the OCTAVE and U-ACHIEVE trials, highlighting TOF's durability and UPA's immediate effectiveness [7,8,11,24]. Despite its shorter observation period and smaller sample size, UPA achieved a remission rate of 47% at week 8 among Bio/JAKi failure sessions, emphasizing its rapid onset of action, as shown in clinical trials and network meta-analyses [11,20]. Additionally, it maintained a remission rate of 50% at week 48, suggesting potential sustained efficacy. However, UPA's long-term efficacy requires further exploration in diverse clinical settings. In our regional hospital network, UPA and, to a lesser extent, TOF were more frequently used after multiple prior biologics, indicating potential confounding by indication that can lower absolute remission despite UPA's known utility in refractory disease. Given the modest sample and event counts, we did not apply propensity or multivariable adjustments to avoid overfitting; findings should therefore be interpreted as pragmatic estimates rather than adjusted causal effects.

The adverse events reported in our study align with the known safety profiles for JAKis. Herpes zoster is a recognized risk of JAKi administration [8]. Although we only observed it with TOF, the absence of herpes zoster events in the FIL and UPA groups likely reflects shorter observation periods and smaller sample sizes rather than intrinsic safety differences. Rare but serious events like PCP and interstitial pneumonia were observed, but not specific to any JAKi. Although PCP during TOF or UPA therapy has been reported [29,30], the limited data and lack of systematic studies make causality unclear. However, the case of interstitial pneumonia observed in our study raises concerns. These findings underscore the need for vigilant monitoring and further study of JAKi adverse events.

Collectively, the favorable remission rates observed for FIL in Bio/JAK-naïve sessions support its consideration as an initial therapeutic option. After Bio/JAKi treatment has failed, however, TOF may be more appropriate for patients with stable disease who require sustained remission, while UPA could be prioritized for those presenting with severe active symptoms that demand rapid intervention. By selecting the JAKi based on clinical severity, therapeutic goals, and comparable safety observations, this strategy may optimize outcomes even in refractory UC.

This study has several limitations. Its retrospective design introduced biases because of variability in treatment selection, follow-up practices, and timing of assessments and treatment discontinuation. In addition, the lack of standardized remission criteria limits the study's generalizability. Furthermore, the small UPA sample size and shorter observation periods for both FIL and UPA reduce the reliability of long-term comparisons. Consistent observation periods in future studies would improve comparability. Finally, we did not record data regarding biomarkers such as leucine-rich alpha-2 glycoprotein and platelets owing to database limitations, which may have restricted the identification of predictors of remission. Comparisons were unadjusted; propensity score or multivariate analyses were not performed because of the limited sample size and events, so residual confounding cannot be excluded.

## Conclusions

Our real-world data suggest that FIL may be a useful option for Bio/JAKi-naïve patients and remained effective after prior failures, suggesting broad applicability. UPA and TOF showed similar remission rates in our cohort. These findings may assist clinicians in treatment selection in real-world practice.
